# Diversity, Community, and Function of Lactic Acid Bacteria in the Brewing of Strong-Flavor Baijiu: A Review

**DOI:** 10.3390/foods15142484

**Published:** 2026-07-14

**Authors:** Liwei Wang, Yanfei Xiong, Ping Song, Bo Deng, Wenjun Nie, Bin Lian

**Affiliations:** 1 School of Food Science and Pharmaceutical Engineering, Nanjing Normal University, Nanjing 210023, China; 242702025@njnu.edu.cn (L.W.); songping@njnu.edu.cn (P.S.); 2 National Engineering Research Center of Solid State Brewing, Luzhou 646000, China; xiongyf@lzlj.com (Y.X.); dengbo@lzlj.com (B.D.); 3 School of Life Sciences, Nanjing Normal University, Nanjing 210023, China; 221201029@njnu.edu.cn; 4 College of Marine Science and Engineering, Nanjing Normal University, Nanjing 210023, China

**Keywords:** strong-flavor Baijiu, lactic acid bacteria, species diversity, function, community succession, diversified applications

## Abstract

Lactic acid bacteria (LAB) are the core functional microbes in strong-flavor Baijiu brewing. Through lactate metabolism, they maintain the ecological balance of the fermentation pit, regulate the synthesis of organic acids and esters, and thus critically influence flavor compound formation and quality stability. Current research focuses on screening functional LAB strains and analyzing fermentation mechanisms and metabolic networks, yet challenges remain regarding complex species lineage, unclear ecological interaction mechanisms, and difficulties in dynamic regulation. This review summarizes LAB diversity in the brewing system, their metabolites and regulation, community interaction networks, niche advantages, and diversified applications. Specifically, it investigates LAB species in distinct ecological niches such as fermented grains (Jiupei), pit mud, and Daqu; clarifies metabolic pathways and products and their associations with flavor substances; and evaluates dynamic changes in LAB during brewing and interactive mechanisms with other fermentative microorganisms. In addition, the review presents findings on probiotic functions of LAB, their applications in food preservation, and their roles in environmental protection, and proposes future research directions. The aim is to provide novel insights for functional utilization of LAB in the brewing system and quality enhancement of Baijiu, as well as a reference for broader and diversified applications.

## 1. Introduction

Baijiu, as a quintessential example of a traditional Chinese distilled spirit, plays an essential role in the national economy and in preserving Chinese culture [1]. The production of Chinese Baijiu is an intricate process reliant primarily on microbial communities in fermentation pits, and its distinctive profile is ultimately shaped through a series of stages, including solid-state fermentation, distillation, ageing, and blending [2]. Based on aromatic characteristics and production methods, Chinese Baijiu can be classified into types such as strong-flavor, sauce-flavor, light-flavor, and blended-flavor Baijiu [3]. Among these, strong-flavor Baijiu is widely popular due to its harmonious flavor and rich aroma; its market share has long remained stable at approximately 50%, making it the leading category among all types of Baijiu [4]. However, the makers of strong-flavor Baijiu also face numerous challenges: conventional brewing methods rely heavily on the microbial diversity in aged fermentation pits and Daqu, as well as on stability of functional strains, which directly affect the quality and yield of the spirit [5]; meanwhile, the fermentation process is influenced by multiple factors, including raw materials, the environment, and human intervention, which readily lead to fluctuations in product quality [6,7]. In recent years, consumers have become increasingly discerning with regard to the quality and health benefits of Baijiu, creating an urgent need for a thorough understanding of how functional microbial communities affect the quality of the liquor during fermentation. Against this backdrop, lactic acid bacteria—as a key functional microbial group in the brewing environment—have attracted widespread attention from both industry and academe.

Lactic acid bacteria (LAB), as a group of Gram-positive bacteria that ferment sugars to produce lactic acid [8], are characterized by high diversity and tolerance to low pH, high salinity, and high temperature [9,10]. Research into LAB began in the 19th century [11], when their defining characteristic—the ability to inhibit the growth of spoilage bacteria—was discovered [8]. This has led to their widespread use in fermenting foods, such as yoghurt, cheese, and kimchi, as well as in prolonging the shelf life of food products [12,13,14,15]. Subsequent research indicated that LAB can act as potential probiotics, regulating the gut microbiota and enhancing the body’s immune function [16]. In recent years, advances in molecular biology and genomics have enriched our understanding of the metabolic pathways, functional characteristics, and host-interaction mechanisms of LAB. LAB also constitute one of the dominant microbial communities in the fermentation of strong-flavor Baijiu [17]. The lactic acid they produce serves as a precursor for the synthesis of key flavor compounds such as ethyl lactate [9]. Besides LAB, the brewing ecosystem harbors diverse functional groups, including saccharifying molds (e.g., *Aspergillus*, *Rhizopus*), ethanol-producing yeasts (e.g., *Saccharomyces*, *Pichia*), aroma-producing Bacillus, and caproic acid-producing anaerobes (e.g., *Clostridium*, *Caproiciproducens*), which together form a complex cross-kingdom network that drives starch hydrolysis, alcohol fermentation, and ester synthesis. Meanwhile, LAB create an acidic environment through acid production, thereby suppressing acid-sensitive contaminating bacteria in the fermentation environment and regulating interactions with other functional microorganisms [18]; furthermore, their growth exerts minimal influence on yeast and ethanol production, which is conducive to the formation of flavor compounds such as acids, alcohols, and esters [19]. LAB can not only directly shape the flavor and quality of Baijiu but also help regulate the structure of the microbial community in the fermented grains and the fermentation environment, thereby playing a decisive role in the production of strong-flavor Baijiu. However, the functional mechanisms of LAB in brewing environments remain largely unknown. Specifically, the strain lineages in the fermentation environment are complex, and the functional differences between species and genera remain unclear; the interactive networks with other microbial communities have yet to be elucidated; there is a lack of systematic research into the dynamic regulatory mechanisms of metabolic functions; and in situ monitoring techniques are lagging in terms of their development and applicability, making it difficult to support precise regulation. These issues severely hinder the utilization of the functions of LAB and improvements in the quality of Baijiu.

This review summarizes recent research progress on the species diversity of LAB in the fermentation of strong-flavor Baijiu, their metabolic pathways and relationship with flavor compounds, community succession and interactive networks, as well as their diversified applications. The review is aimed at identifying key bottlenecks in current research and providing foundational data and new insights for the quality control of strong-flavor Baijiu and the innovative application of LAB-related products.

## 2. LAB and Species Diversity During the Fermentation of Strong-Flavor Baijiu

In traditional taxonomy, LAB can be classified across several families and genera, such as *Lactobacillaceae* and *Streptococcaceae*. In 2020, Zheng [20] conducted a taxonomic assessment of the *Lactobacillaceae* and *Leuconostocaceae* based on whole-genome sequences (without involving *Streptococcaceae*) and merged these two families into a new family, *Lactobacillaceae*. In this reclassification, the former genus *Lactobacillus* was split and reclassified into 25 genera, including the emended genus *Lactobacillus*, *Paralactobacillus*, and 23 novel genera such as *Lacticaseibacillus*. Meanwhile, the genera originally classified in *Leuconostocaceae*, including *Leuconostoc*, *Weissella*, *Pediococcus*, *Fructobacillus*, and *Oenococcus*, were also incorporated into this merged new family [21]. This taxonomic update underpins our understanding of the diversity and functional differentiation of LAB within the strong-flavor Baijiu brewing ecosystem.

With regard to the fermentation ecosystem of strong-flavor Baijiu, some scholars have systematically investigated the LAB species present in different ecological niches, such as fermented grains, pit mud, and Daqu. Su [22] found that LAB were the most abundant group of microorganisms in the fermented grains from a single fermentation pit; there were a total of 49 species detected, with dominant LAB (relative abundance > 0.1%), including *Levilactobacillus brevis*, *Companilactobacillus crustorum*, *Latilactobacillus curvatus*, *Lactiplantibacillus pentosus*, *Lactococcus lactis*, *Leuconostoc citreum*, *Weissella confusa*, and *Weissella cibaria*. Qian [23] discovered that the abundance of LAB in the fermented grains of strong-flavor Baijiu is significantly higher than that in pit mud, with the main groups, including *Lactobacillus*, *Weissella*, *Lactococcus* and *Enterococcus*. Furthermore, *Fructilactobacillus sanfranciscensis* was successfully screened and identified in the fermented grains of strong-flavor Baijiu [24]. LAB in the fermented grains are primarily involved in the metabolism of multiple sugars, accumulating lactic acid as a substrate for the synthesis of flavor compounds, such as butyric acid and caproic acid.

Wang [25] isolated and identified *Lacticaseibacillus zeae*, *L. pentosus*, and *Pediococcus acidilactici* from high-quality strong-flavor pit mud. Shen [26] isolated 15 strains of LAB from strong-flavor pit mud, all of which were identified as *Lacticaseibacillus paracasei*. Hu [27] isolated four species of LAB from pit mud, including *Bacillus coagulans*, *Bacillus amyloliquefaciens*, *Clostridium tyrobutyricum*, and *Clostridium butyricum*. To date, 44 strains of LAB belonging to 16 genera and 27 species have been isolated from the pit mud of Luzhou Laojiao [28]. LAB in the pit mud can produce lactic acid and participate in esterification reactions to generate ethyl lactate, while simultaneously inhibiting the growth of contaminating bacteria by maintaining a highly acidic environment. These studies have laid the foundation for investigating the functional microorganisms in pit mud, as well as the domestication and stabilization of LAB [29].

LAB are the dominant bacterial group during the ageing of Daqu; the predominant genera include *Weissella*, *Limosilactobacillus*, and *Lactococcus* [30]. Wang [30] isolated *Lc. lactis* and *Pediococcus pentosaceus* from medium- to high-temperature Daqu. Su [22] discovered that *Weissella hellenica* and *Leuconostoc mesenteroides* are dominant LAB (relative abundance > 0.1%) unique to the Daqu of strong-flavor Baijiu. Recent research has isolated 15 LAB strains from Luzhou Laojiao Daqu, belonging to eight genera and 10 species [28]. Daqu for strong-flavor Baijiu also encompasses a wide variety of LAB, which exerts a key regulatory effect on the metabolism of Daqu polysaccharides and amino acid synthesis, and are closely associated with the formation of higher alcohols and nitrogenous flavor compounds [31].

In summary, the fermented grains, pit mud, and Daqu of strong-flavor Baijiu are all home to rich communities of LAB with diverse structures and functions (Table 1). However, current research remains predominantly focused on isolation, culture, and amplicon sequencing, and there is a lack of sufficient understanding regarding the colonization, migration, and evolution of LAB across different ecological niches. With the advances in analytical and DNA detection techniques, real-time quantitative PCR and next-generation sequencing can be leveraged to monitor the abundance and dynamic changes in LAB during the fermentation of strong-flavor Baijiu [28]. This will lead to the discovery of further LAB resources, providing a theoretical basis for the targeted regulation of the fermentation process and enhancement of the quality of Baijiu.

Although high-throughput sequencing has revealed rich LAB diversity in the fermentation system of Chinese strong-flavor baijiu, existing studies have notable methodological limitations. First, there is a marked discrepancy between sequencing data and culturable strains: for example, Su et al. [22] found that *We. confusa* showed high relative abundance in fermented grains via amplicon sequencing, but it was difficult to obtain pure cultures upon isolation; conversely, some low-abundance species in sequencing, such as *Ls. paracasei*, were readily isolated [26]. This contradiction suggests that inferring ecological functions solely from relative abundance may be misleading. Second, the lack of standardized sampling protocols and a unified comparative framework across studies makes it unclear whether the observed differences in LAB composition across production regions and cellar ages arise from geographical factors or technical biases. Consequently, current diversity data are mostly static snapshots, and the migration, colonization dynamics, and evolutionary trajectories of LAB among the three ecological niches (fermented grains, pit mud, and Daqu) remain largely unexplored.

## 3. Metabolic Pathways of LAB and Their Relationship with Flavor Compounds in Strong-Flavor Baijiu

LAB drive the formation of flavor compounds in strong-flavor Baijiu through various sugar metabolism pathways, and the analysis of their metabolic networks is the theoretical basis for achieving precise regulation of the brewing process.

### 3.1. Main Metabolic Types of LAB

Based on the differences in metabolic pathways and fermentation products, LAB can be primarily classified into homofermentative and heterofermentative types [32]; facultatively heterofermentative LAB and bifidobacteria-type fermentative bacteria are also included. It is worth noting that a unique lactic-acid-producing pathway, known as the bifidobacterial shunt (or phosphoketolase pathway), exists in the genus *Bifidobacterium*. However, *Bifidobacterium* belongs to the phylum Actinobacteria, whereas LAB are classified within the phylum Firmicutes (family *Lactobacillaceae*) [20]. Therefore, *Bifidobacterium* is taxonomically distinct from LAB and is not considered part of this functional group. However, because Bifidobacteria can produce lactic acid, their functions and applications highly overlap with those of LAB, and they are often regarded as “similar LAB”.

Homofermentative LAB, represented by *L. delbrueckii* and *E. faecalis* [33], metabolize glucose via the Embden–Meyerhof–Parnas (EMP) pathway, producing lactic acid as the sole product; theoretically, each mole of glucose yields two moles of lactic acid [34]. This pathway begins with the phosphorylation of glucose to glucose-6-phosphate, which is subsequently converted to pyruvate through sequential catalysis by key enzymes such as phosphofructokinase, fructose-bisphosphate aldolase, and glyceraldehyde-3-phosphate dehydrogenase. Consequently, lactate dehydrogenase (LDH) reduces pyruvate to lactic acid and regenerates nicotinamide adenine dinucleotide (NAD^+^). The pathway is regulated by the intracellular adenosine triphosphate and adenosine diphosphate and nicotinamide adenine dinucleotide/NAD^+^ balances [35,36,37]. The expression of the LDH gene is modulated by oxygen: it is upregulated under hypoxic conditions to promote the fermentation of lactic acid, and downregulated under aerobiosis, shifting metabolism towards respiratory [35].

Heterofermentative LAB, represented by *Leuconostoc*, *Oenococcus*, and certain *Lactobacillus* strains, primarily utilize the phosphoketolase (PK) pathway, which varies depending on the substrate: when growing on pentose sugars, they directly enter this pathway; when the substrate is hexose, due to the lack or low expression of fructose-bisphosphate aldolase, they must rely on the PK pathway [33,38]. Glucose must first be oxidized to 6-phosphogluconate, which is then decarboxylated by 6-phosphogluconate dehydrogenase to release one CO_2_ and form ribulose-5-phosphate, and subsequently cleaved by PK into glyceraldehyde-3-phosphate and acetyl phosphate, with the theoretical maximum yield of lactic acid limited to one mole per mole of glucose [34]. Additionally, the excess NAD(P)H generated during this process is transferred to acetyl-CoA, which is then reduced to ethanol [38]. Consequently, the main products of heterofermentative LAB are lactic acid, ethanol, and CO_2_, accompanied by by-products such as diacetyl, formic acid, acetoin, and acetic acid [39].

Facultatively heterofermentative LAB, such as *L. plantarum* and *L. casei*, possess complete EMP and PK pathway enzyme systems in their genomes: when using hexoses such as glucose, they metabolize via the EMP pathway to produce almost exclusively lactic acid; when the substrate is composed of pentoses, they activate the PK pathway, mainly generating lactic acid and acetic acid [40].

**Figure 1:**
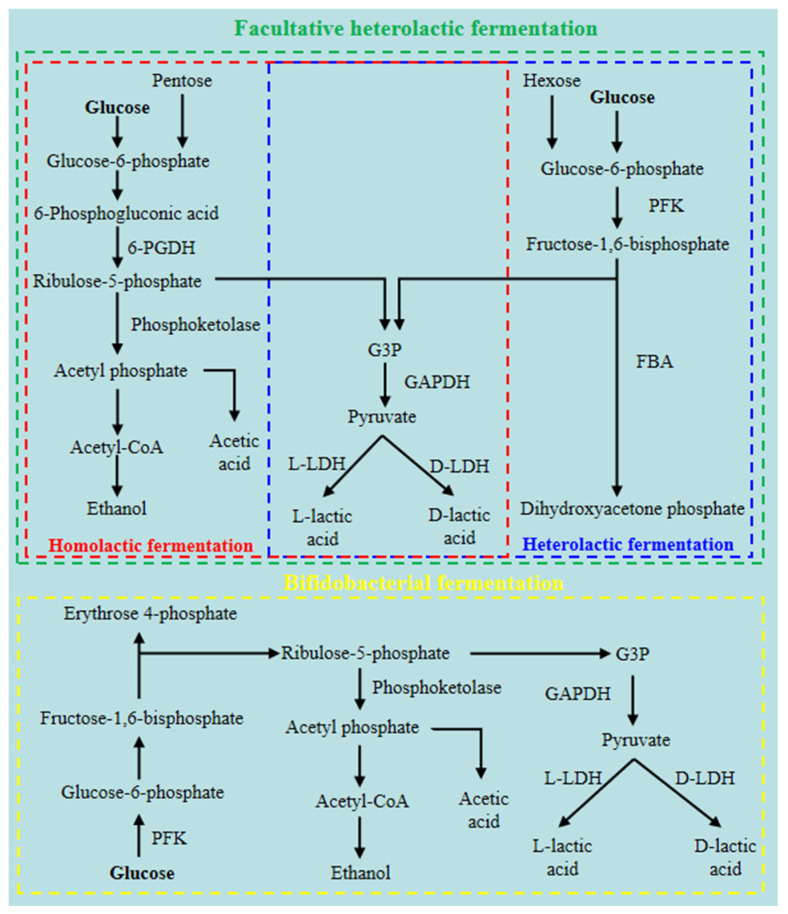
Lactic acid-producing metabolic pathways. (PFK: phosphofructokinase; FBA: fructose-1,6-bisphosphate aldolase; G3P: glyceraldehyde-3-phosphate; GAPDH: glyceraldehyde-3-phosphate dehydrogenase; 6-PGDH: 6-phosphogluconate dehydrogenase).

Bifidobacteria possess a unique fermentation pathway: via the hexose phosphate dehydrogenase pathway, one mole of glucose can be converted into one mole of lactic acid and 1.5 moles of acetic acid [9]. The process is described as follows: glucose is first converted to fructose-1,6-bisphosphate by enzymes such as phosphofructokinase, which is then cleaved into erythrose-4-phosphate and ribulose-5-phosphate; ribulose-5-phosphate is subsequently cleaved by PK into glyceraldehyde-3-phosphate and acetyl phosphate. Glyceraldehyde-3-phosphate then enters the lower part of the EMP pathway to generate pyruvate, which is reduced to lactic acid by LDH [41]. The four lactic acid-producing metabolic pathways mentioned above are illustrated in Figure 1.

It is noteworthy that although all four metabolic pathways exist in microorganisms, in the brewing system of strong-flavor Baijiu, LAB produce lactic acid mainly through homofermentation, heterofermentation, and facultative heterofermentation. The bifidobacteria pathway is found only in the genus *Bifidobacterium*, whose abundance is extremely low in fermented grains, pit mud, or Daqu; its contribution to the production of lactic acid during the fermentation of Baijiu is therefore negligible. This finding implies that research into lactic acid metabolism in strong-flavor Baijiu should focus solely on the three aforementioned fermentation pathways and their regulatory conditions, without incorporating the bifidobacteria pathway into the analytical framework. Failure to do so may lead to wasted research resources or biased conclusions. From a microbial ecology perspective, this also confirms that *Bifidobacterium* is not a dominant group in this brewing system, and its metabolic activities exert a negligible influence on the acidity and flavor formation of the fermented grains.

### 3.2. Metabolites of LAB and Their Association with the Strong-Flavor Baijiu

LDH is a key enzyme converting pyruvate to lactic acid in LAB, and its stereospecificity determines the configuration of lactic acid: D-lactic acid and L-lactic acid are generated by D-LDH and L-LDH, respectively [32]. In humans and other eukaryotes, lactic acid exists predominantly in the L form [42], whereas D-lactic acid is primarily derived from LAB [43]. Xu [44] found that D-lactic acid contributes more to the aroma of strong-flavor Baijiu than L-lactic acid, and that the content of D-lactic acid is generally higher. Fan [45] further revealed that under anaerobic conditions, *L. ozensis* and *Lentilactobacillus diolivorans* in strong-flavor Baijiu mainly produce D-lactic acid, whereas L-lactic acid is mainly synthesized by *Torulaspora delbrueckii*, *L. ozensis*, and *Ac. acetotolerans*. Although D-lactic acid contributes more to the flavor of Baijiu, its regulatory mechanism of synthesis has yet to be revealed.

In addition to lactic acid, LAB also synthesize organic acids, short-chain fatty acids (SCFAs), exopolysaccharides (EPSs), and bacteriocins [46]. Among these, organic acids can significantly lower the environmental pH and inhibit the growth of pathogenic microorganisms [46]. Some LAB also produce unsaturated fatty acids and hydroxy unsaturated fatty acids, helping enhance the host’s defense against pathogenic fungi [10]. Furthermore, LAB can synthesize vitamins, including folate and riboflavin [32]. The bacteriocins produced by LAB are proteins or peptides with broad-spectrum antimicrobial activity [47], offering potential as natural alternatives to chemical preservatives in food preservation. Meanwhile, EPS produced by LAB not only serves as food thickeners and stabilizers but also has health-promoting functions, such as immunomodulation, antioxidant activity, cholesterol-lowering, and antitumor effects [48].

LAB shape the core flavor of strong-flavor Baijiu through diverse metabolic activities. During fermentation, the relative abundance of *Lactobacillus* has a significant positive correlation with the synthesis of lactic acid [45]. Lactic acid is the primary organic acid contributing to the full-bodied mouthfeel of Baijiu, accounting for over 20% of the total organic acid content [49]. It can react with ethanol via chemical esterification to form ethyl lactate [50,51], which imparts a unique fruity and honey-like flavor and is a key component contributing to the richness and persistence of the flavor of Baijiu [52]. Mechanistically, ethyl lactate is formed via esterification of lactic acid and ethanol, a reaction that can be catalyzed by esterases produced by certain LAB strains (e.g., *L. plantarum*). However, the dominant route in Baijiu is likely non-enzymatic chemical esterification, which is kinetically favored at low pH and high ethanol concentration. For caproic acid, LAB do not synthesize it directly; instead, they supply lactate as a precursor for *Caproiciproducens* and related clostridia, which convert lactate to caproate via the reverse β-oxidation pathway involving key enzymes, such as acyl-CoA dehydrogenase and β-hydroxyacyl-CoA dehydratase. Furthermore, heterofermentative LAB produce diacetyl from α-acetolactate through oxidative decarboxylation, contributing buttery notes, though this pathway is suppressed under strictly anaerobic conditions. Recent integrated metagenomic and metabolomic studies have revealed the stage-dependent molecular basis of ester biosynthesis during pit fermentation [53]. Three distinct phases were identified: during days 0–15, fungi (*Saccharomyces*, *Aspergillus*, *Rhizopus*) dominate, with high esterase (EST) gene abundance driving the synthesis of ethyl acetate and ethyl butyrate. In phase 2 (days 15–35), acidogenic LAB (*Acetilactobacillus* > 64%) proliferate, and only ethyl lactate shows a marked increase, associated with 42 EST genes. In phase 3 (days 35–117), a *Lactobacillus–Acetilactobacillus* consortium (>74%) becomes the core driver of rapid ester accumulation, with high EST gene abundance directly linked to the synthesis of ethyl caproate and ethyl butyrate. Furthermore, bacteria of the genus *Lactobacillus* possess genes related to the synthesis of diverse flavor compound precursors and are significantly associated with the production of at least seven esters, including ethyl phenylacetate, ethyl hexanoate, and ethyl butyrate, as well as phenylacetic acid [54]. LAB also function in the production of amino acids and polysaccharide metabolism during Baijiu fermentation, contributing to the generation of amino acids and reducing sugars. These substances serve as precursors for the further formation of important flavor compounds, such as higher alcohols, furfural, pyrazines, and furans [9]. However, lactic acid plays a dual role in flavor shaping. An appropriate amount of lactic acid can optimize the flavor and suppress the muddy off-flavor [55]; however, excessive lactic acid disrupts the flavor balance, resulting in an imbalance in the ratio of ethyl hexanoate to ethyl lactate, inducing a sour and astringent taste, weakening the body of the liquor, and ultimately compromising the flavor quality of Baijiu [56,57].

Although the metabolic network of LAB has been relatively well investigated, the functional analysis of LAB in the fermentation and regulation of strong-flavor Baijiu still faces challenges. The primary contradiction lies in the significant discrepancy between “pure culture models in the laboratory” and “actual multi-species competitive environments”. For instance, although D-lactic acid contributes more favorably to flavor, it remains unclear how LAB maintain their metabolic advantage amid competition with yeasts, molds, and other microorganisms in fermentation processes. Are the syntheses of LAB metabolites sufficiently induced under brewing conditions? Is their production sufficient to impart significant ecological or flavor-regulating functions? Future research should adopt approaches such as synthetic microbial consortia and in situ metabolomics to validate and quantify the metabolic functions of LAB under conditions that closely mimic real brewing environments.

Notably, the fermentation parameters (e.g., initial pH, moisture content, and stacking temperature) vary considerably across different production regions (e.g., Luzhou vs. Yibin vs. northern China). However, the current literature lacks direct controlled experiments comparing these regional factors. For instance, a higher initial moisture content tends to promote heterofermentative LAB, whereas a lower pH favors homofermentative species. Without a unified comparative dataset, it remains unclear whether the observed differences in LAB communities between regions are genuine ecological responses or simply artifacts of distinct sampling protocols. We advocate for future multi-center trials using standardized model fermentation systems to disentangle the effects of geographical factors from technological biases.

## 4. Succession in the LAB Community and Its Interaction with Other Microorganisms in the Fermentation of Strong-Flavor Baijiu

LAB are deeply integrated in a microbial interaction network comprising bacteria, yeasts, and molds during the fermentation of strong-flavor Baijiu. Their dynamic succession and metabolic synergy can directly determine the fermentation efficiency of the fermented grains, the profile of flavor compounds, and ultimately the quality of the Baijiu.

### 4.1. Colonization and Stress-Resistance Mechanisms of LAB in the Fermentation of Strong-Flavor Baijiu

**Figure 2:**
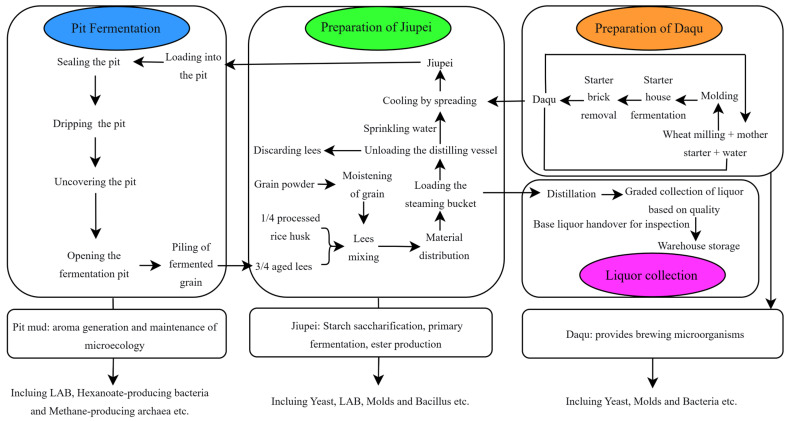
The brewing process of strong-flavor Baijiu and the function of LAB.

Conventional solid-state fermentation of strong-flavor Baijiu is an open, multi-species co-fermentation system that relies on the synergistic effects of Daqu (for saccharification and fermentation), cereal raw material conversion, and pit mud for aroma production [58] (Figure 2). This creates an extreme environment characterized by high acidity, high ethanol concentration, and high osmotic pressure, which is therefore lethal to most microorganisms [59]. LAB are capable of colonizing fermented grains, pit mud, and Daqu and show robust proliferation under such conditions due to their diverse stress resistance mechanisms.

To withstand high ethanol stress, LAB regulate membrane fatty acid composition (increasing unsaturated fatty acids) and fluidity, and coordinate energy metabolism, antioxidant systems, and biofilm formation, forming a multi-layered protective network that sustains growth and metabolic homeostasis [60]. Surface adhesion proteins and EPS of LAB promote initial attachment at the Jiupei–pit mud interface, and biofilm formation confers long-term colonization ability in the solid matrix [61].

Regarding the mechanisms underlying acid resistance, LAB possess multiple acid resistance-related genes, whose expression patterns vary with pit age and fermentation time. In a 20-year-old pit, *argR*, *ilvE*, *cfa*, *gshA*, and *DnaK* were positively correlated with acid resistance at the early fermentation stage, whereas in a 5-year-old pit, *aspA*, *ilvE*, *cfa*, and *argR* showed early-stage correlation, and *DnaK* was correlated thereafter [59]. Upon exposure to acidic conditions, LAB mount a complex stress response marked by upregulation of acid resistance genes, including the F_0_F_1_-ATPase system and chaperone proteins that stabilize cellular proteins at low pH. This response not only enhances survival but also improves intercellular communication and resource sharing through quorum sensing, particularly via the AI-2 signaling pathway [62,63]. Under osmotic stress conditions, certain LAB exhibit salt tolerance, enabling them to endure high osmotic pressure and ferment diverse carbon sources under high salt conditions [64].

Beyond coping with extreme environments, the metabolic activities of LAB also participate in pit mud ripening. Jiao [65] found that with increasing pit age, the contents of total iron and crystalline iron minerals significantly decreased, while the Fe^2+^/Fe^3+^ ratio and the content of amorphous iron (hydr) oxides significantly increased. This change is directly correlated with the metabolic activities of LAB, which can reduce Fe^3+^ to Fe^2+^ under acidic conditions [66]. Furthermore, the antimicrobial substances synthesized by LAB play an essential role in maintaining microecological stability. Lactic acid, other organic acids, hydrogen peroxide, and bacteriocins produced by LAB all show antimicrobial activity; among these, bacteriocins are effective against both Gram-positive and Gram-negative bacteria [67]. Bacteriocins from a strain of *Ls. paracasei* isolated from Luzhou Laojiao pit mud inhibit harmful bacteria without influencing mold and yeast proliferation [68]. This characteristic provides a potential tool for the selective regulation of the brewing microbial community. Future efforts will enhance LAB colonization and stress resistance through adaptive evolution or synthetic biology, potentially developing them into microbial regulators for targeted improvement in the flavor of Baijiu.

Despite the promising probiotic and preservative properties of LAB isolated from Baijiu, translating these findings into industrial products faces several hurdles. First, many strains exhibit poor growth and metabolite yields under standardized laboratory media compared to the complex brewing environment, necessitating adaptive evolution or CRISPR-based engineering to enhance robustness. Second, safety concerns, such as the presence of virulence genes or biogenic amine production, must be systematically assessed using whole-genome sequencing and phenotypic tests before any food or feed application. Third, the economic feasibility of bacteriocin or EPS production is questionable, as downstream purification costs often outweigh the benefits over conventional chemical preservatives; in situ fermentation or immobilization techniques may offer partial solutions. Finally, regulatory approval (e.g., GRAS status) requires extensive toxicological data, which currently are lacking for Baijiu-derived strains. Therefore, future research should prioritize strain safety screening, cost-effective production processes, and pilot-scale validation.

### 4.2. Dynamic Changes in LAB and Their Interactions with Other Microorganisms in the Fermentation of Strong-Flavor Baijiu

In the solid-state fermentation of strong-flavor Baijiu, Daqu serves as the saccharifying and fermenting agent and is mainly produced from wheat [69]. During Daqu fermentation and storage, *Lactobacillus* is one of the dominant bacterial groups [70]; however, as the temperature increases rapidly during fermentation, its relative abundance decreases significantly [71]. During aging, the relative abundance of LAB fluctuates: it first decreases, then increases, and finally decreases again. After one month of storage, the abundance thereof rises significantly; after two to three months, it gradually falls to its lowest level; and from the fourth to sixth month, it continues to decline yet remains above 50% [30]. Overall, *Lactobacillus* remains the dominant bacterial genus from the end of fermentation through the storage period [72]. As Daqu is moved from the culturing room to the aging room, and until use, the microbial community shifts: *Bacillus* gradually becomes dominant, while the proportion of *Lactobacillus* decreases [70].

Fermented grains serve as the primary habitat for microorganisms and the direct source of flavor compounds in strong-flavor Baijiu production [73,74]. Although the proportions of dominant genera vary across different layers of fermented grains, the overall differences are minor. Within a single layer, *Bacillus* dominates early, whereas *Lactobacillus* becomes absolutely dominant at the middle and late stages [75]. As an example, Su [22] reported the following dynamic changes in LAB in fermented grains during the fermentation of strong-flavor Baijiu: on the day of pit entry, the relative abundance of LAB peaked at 51.7%; one day after sealing the pit, it plummeted to 11.2%. From Day 1 to Day 7, LAB proliferated rapidly, leading to a sharp increase in abundance. After Day 7, the increase slowed, reaching 78.8% by the end of fermentation, re-establishing the dominance of LAB. However, the reported successional patterns of the LAB community are not entirely consistent across different studies. For example, some studies have found that the relative abundance of LAB exceeded 50% on the day of pit entry [22], while others have indicated that *Bacillus* dominated during the early fermentation stage, with LAB abundance remaining below 30% [75]. Such discrepancies may arise from seasonal variations in raw material composition, initial acidity of the fermented grains, or Daqu quality; however, a systematic comparative analysis of these confounding factors is lacking in the existing literature.

Strong-flavor Baijiu employs a distinctive “solid state fermentation in pit mud” process [76]; pit sealing creates an anaerobic environment that blocks external microorganisms and harnesses functional microorganisms enriched in the pit mud for aroma production [77,78]. At the early stage of fermentation, fungi and bacteria derived from Daqu are dominant; at the middle stage, a sharp succession occurs, with *Acetilactobacillus* becoming the dominant genus (64.13%); at the late stage, a combined community dominated by *Acetilactobacillus* and *Lactobacillus* forms, with a total relative abundance of 74.53% (Table 2) [53]. Meanwhile, in new pit mud, *Lactobacillus* and other genera dominate, but their abundance declines with the increasing age of the pit. In contrast, genera such as *Clostridium* and *Caproiciproducens* gradually become dominant in aged pit mud, and the community structure stabilizes [29].

**Figure 3:**
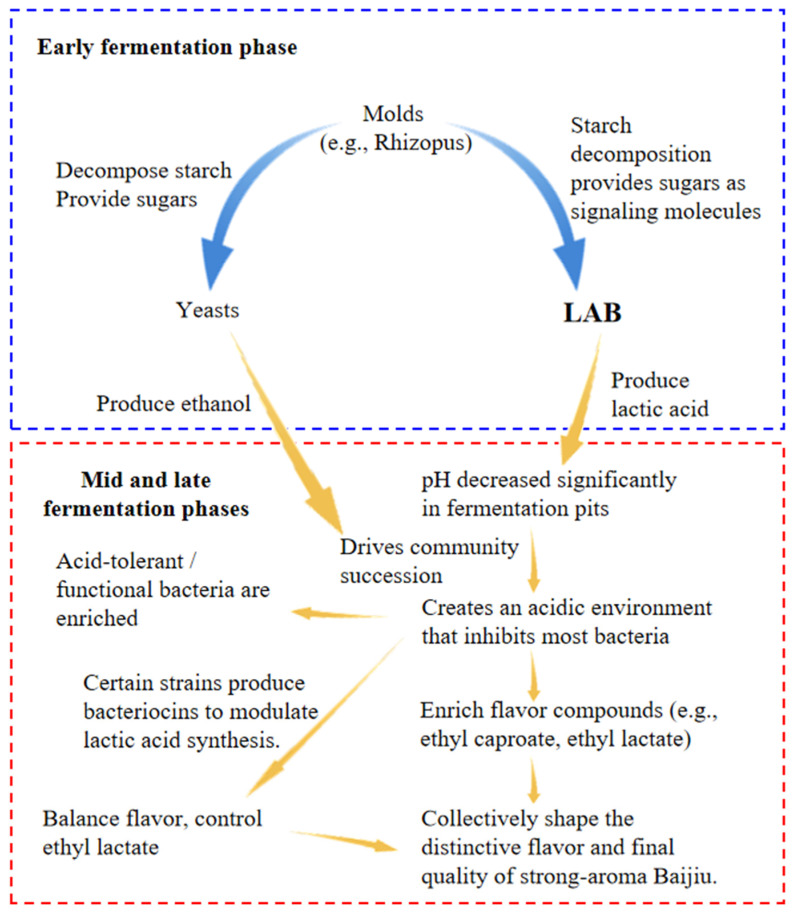
Microbial interactions during the fermentation of strong-flavor Baijiu.

The fermentation of strong-flavor Baijiu is a dynamic process of synergistic metabolism within a microbial community (Figure 3) [79]. To clarify the temporal dynamics, we propose dividing the fermentation into three phases: (i) the early phase (days 0–7), characterized by aerobic activity of Bacillus and mold-derived enzymes, with LAB abundance below 20%; (ii) the middle phase (days 8–25), when oxygen depletion triggers rapid LAB proliferation (up to 70% relative abundance), accompanied by a sharp pH drop; and (iii) the late phase (days 26–60), where LAB stabilize and interact with caproic acid-producing bacteria (e.g., *Caproiciproducens*) to convert lactate to caproate. As key biomarkers of community succession, LAB can influence the quality of Baijiu through their metabolic activities [80,81]. In the late fermentation stage, LAB abundance is strongly positively correlated with acidity, confirming LAB as a key driver of shifts in the bacterial community [58,81]. Especially during the secondary temperature rise, rapid accumulation of LAB and the resulting acidic environment suppress many acid-sensitive bacteria, markedly reducing bacterial diversity [82]. Meanwhile, lactic acid also serves as a substrate for other functional bacteria. Jin [83] found that *Caproiciproducens* in the pit mud of strong-flavor Baijiu can convert lactic acid in yellow water into caproic acid, thereby offering a new strategy for increasing ethyl caproate.

LABs are also tightly coupled with the fungal community. Yeasts persist throughout fermentation [19], converting sugars into ethanol and CO_2_ while also producing small amounts of higher alcohols and esters [84,85]. During fermentation of Baiju, yeasts and *Lactobacillus* exhibit a significant positive correlation, forming metabolic complementarity: yeasts dominate alcohol production, while *Lactobacillus* further converts some of the ethanol into acids and synergistically participates in the formation of esters [58]. Furthermore, LAB also have positive interactions with molds during the saccharification stage. Rhizopus is significantly positively correlated with Lactobacillus, and benzyl alcohol produced by *R. microsporus* promotes the growth of *L. fermentum* [86]. Molds break down starch into fermentable sugars at the early stage of fermentation, providing basic substrates for the subsequent metabolism of LAB and yeasts [87]. Shu [68] discovered that bacteriocins secreted by *Ls. paracasei* can inhibit the growth of certain LAB and reduce the production of lactic acid without influencing molds and yeasts, providing an important reference for controlling the ethyl lactate content.

In summary, LAB regulate environmental physicochemical factors via acid production, directly inhibiting competitors and driving bacterial succession. Concurrently, through metabolic division of labor, substrate provision, and signal exchange, they establish a tightly synergistic network with yeasts and molds that collectively determines the unique flavor and quality of the final product [88]. The interactive mechanisms operating between LAB and the pit mud microecology remain to be fully elucidated, and niche competition challenges the standardized use of functional microbial agents. To address this, a targeted pit mud domestication strategy for pit mud has been developed to promote the efficient conversion of lactic acid into caproic acid [89]. In addition, genome-scale metabolic models, as mathematical frameworks, can delineate microbial metabolic functions and interactions and predict fermentation dynamics [90]; they have already been successfully used to study the metabolic regulation of LAB [91].

Although it is known that there are complex metabolic interactions among LAB, yeasts, Bacillus, and caproic acid-producing bacteria (e.g., *Caproiciproducens*), most existing studies remain at the level of genus- or species-level correlation descriptions and have not yet reached the precision of identifying “which specific species produces which metabolite that mediates such interactions.” For example, lactic acid produced by LAB is considered a substrate for *Caproiciproducens* [83]; however, in the actual fermentation system, the concentration of lactic acid fluctuates considerably, and quantitative threshold studies are currently lacking—that is, to determine exactly when lactic acid promotes caproic acid synthesis and when it conversely inhibits the fermentation system. Moreover, the physical contact dependencies (e.g., biofilm co-aggregation) between LAB and fungi (such as molds and yeasts), or the cross-kingdom communication mediated by signaling molecules (e.g., AI-2), have been scarcely explored in the Baijiu brewing system. Yet these very mechanisms may be the key to unlocking the bottleneck of “difficulty in artificially intervening in the solid-state fermentation process”.

## 5. Diversified Applications of LAB

LAB not only play a key role in the fermentation of Baijiu, but their probiotic functions, the value of food preservation, and cross-field applications have also attracted considerable attention.

### 5.1. Probiotic Functions of LAB and Human Health

As beneficial active microorganisms for animal hosts, LAB are widely used because of their diverse health-promoting effects [92]. LAB also inhabit the human gut, where they perform important physiological and metabolic functions [93], such as regulating the gut microbial ecology and community structure, strengthening the intestinal epithelial barrier, and enhancing intestinal immune responses [94]. Clinical studies confirm that LAB can help alleviate functional defecation disorders, improve diarrhea, constipation, and stool quality, and restore normal defecation patterns [94]. These beneficial effects are mainly ascribed to the lactic acid produced during their metabolism, which reduces intestinal pH and inhibits the growth of pathogenic bacteria. In addition, LAB can competitively exclude pathogens through niche occupation, while also activating the gastrointestinal immune system and maintaining intestinal microecological balance [95]. Nie [96] co-fermented LAB with *Flammulina velutipes* and fed the fermentation product to mice, evincing that it exerts auxiliary hypolipidemic effects, increases the diversity of gut microbiota, and improves the intestinal metabolic status of hyperlipidemic mice.

In terms of nutritional metabolism, LAB can break down complex food components (e.g., casein) and degrade lactose, thus helping lactose-intolerant individuals digest dairy products [97]. Furthermore, the SCFAs produced by LAB through the metabolism of prebiotics can promote the proliferation of intestinal mucosal epithelial cells, enlarge the microvillar surface area, and facilitate calcium absorption [93]. Notably, LAB produce two isomers of lactic acid: L-lactic acid is the predominant form of lactate metabolized by the human body, whereas D-lactic acid is primarily derived from LAB. Excessive accumulation of D-lactic acid may be associated with gut dysbiosis and related diseases, making it a potential target for therapeutic intervention [42,44]. Recent studies have revealed that certain LAB strains isolated from infant feces can significantly abate urate-induced inflammatory responses and exhibit uric acid-lowering functions [92].

### 5.2. Natural Food Preservation Functions of LAB

Within the market demand for lactic acid, food industry applications account for approximately 85%, primarily as a preservative and acidity regulator [39]. LAB lower the pH by producing lactic acid, thereby inhibiting the growth of pathogenic microorganisms and prolonging the shelf life of food products [97].

The antimicrobial effects of LAB primarily originate from the bacteriocins and organic acids generated during their metabolism. Based on molecular weight, LAB produce three classes of bacteriocins [98]: Class I (lantibiotics) consist of one or two small peptides (<5 kDa) and exhibit inhibitory activity against foodborne pathogens and Gram-negative bacteria; Class II bacteriocins (small non-lantibiotics) are small peptides of 30–60 amino acids (<10 kDa) that disrupt bacterial cell membranes, ultimately leading to cell death; Class III bacteriocins (large non-lantibiotics) have molecular weights exceeding 30 kDa and are capable of lysing cell membranes [47]. In addition, LAB produce organic acids, such as lactic acid, acetic acid, and propionic acid, as well as other antimicrobial substances containing ethanol and hydrogen peroxide during fermentation [99]. These compounds exert antimicrobial effects by disrupting bacterial cell membrane structures, interfering with intracellular proton gradients, inactivating key enzymes, and inhibiting DNA/RNA synthesis [100]. LAB can also suppress pathogenic and spoilage microorganisms through nutrient competition, such as competition arising for metal ions [97]. Some studies discovered that when co-cultured with other microorganisms, the expression levels of LAB metabolism-related proteins are significantly upregulated. This metabolic regulation enhances their own metabolic rate and nutrient competitiveness, thus further curbing the growth of other strains in the co-culture system [100].

### 5.3. Further Applications of LAB in Other Fields

LAB also demonstrates broad application prospects in agriculture, pollutant toxicity mitigation, and soil remediation. In agriculture, they are widely applied in silage production, where they inhibit harmful microorganisms, improve the nutritional composition of feed, and improve silage quality [101]. As a feed additive, LAB can maintain the balance of the intestinal microbiota in animals, promote nutrient absorption, and enhance immunity. Feed fermented by LAB shows good palatability, high nutritional value, and a long storage life [102]. Certain LAB also exert detoxifying effects on feed raw materials, thereby reducing the risk of disease in livestock and poultry [103]. Currently, LAB have been formulated into microbial agents either alone or in combination with other probiotics, and are widely applied.

In terms of mitigating environmental pollutant toxicity, LAB can alleviate hepatotoxicity and testicular toxicity induced by exposure to microplastics and nanoplastics (MNPs). The underlying mechanisms include elevating excretion of fecal MNPs, repairing intestinal barrier function, modulating gut dysbiosis, and promoting the production of SCFAs [104]. Furthermore, LAB also have alleviating effects against perfluorooctane sulfonate (PFOS). Owing to their potent antioxidant activity and toxin-binding capacity, LAB can increase the content of cecal SCFA and gut tight-junction protein levels in mice, effectively alleviating PFOS-induced toxicity. This positions LAB as a potential alternative intervention strategy [105].

In soil remediation, LAB application has also yielded positive outcomes. For example, the application of LAB to saline-alkali soil reduces soil pH and electrical conductivity, enriches soil microbial diversity, and suppresses crop diseases [106]. For soils contaminated with heavy metals, *L. plantarum* exhibits strong tolerance to cadmium-zinc co-contamination. It enhances the phytoremediation of cadmium and zinc by enhancing the activities of soil enzymes (urease, ALP, β-DG) and plant antioxidant enzymes (POD, CAT, SOD), as well as the biomass of mustard (*Brassica juncea*) and metal translocation from roots to shoots [107]. The application of LAB to mining-contaminated soils can potentially enhance the bioremediation of heavy metal-polluted soils, offering a new option for the ecological management of soils contaminated with toxic metals.

## 6. Conclusions

Diverse LAB species inhabit the system during the fermentation of strong-flavor Baijiu. Through diverse metabolic pathways, they can drive the synthesis of key flavor compounds, such as lactic acid and ethyl lactate, and form a tightly coupled metabolic network with yeasts and molds, collectively shaping the unique style and quality of strong-flavor Baijiu. Meanwhile, LAB achieve colonization and succession in the high acidity, high ethanol brewing environment by relying on stress resistance mechanisms such as biofilm formation, the F_0_F_1_ ATPase system, and quorum sensing. Future efforts should focus on integrating microbiomics, metabolomics, and synthetic biology to develop synthetic microbial community models that closely mimic actual fermentation conditions. Emphasis should be placed on investigating the synergistic and antagonistic relationships between LAB and functional microorganisms, such as ester-producing yeasts and caproic acid-producing bacteria, as well as deciphering the regulatory switch for efficient synthesis of D-lactic acid. Furthermore, artificial intelligence algorithms should be integrated to realize dynamic simulation and early warning of the fermentation process. In addition, in-situ real-time monitoring techniques, such as electronic noses and near-infrared spectroscopy, should be developed. A synergistic regulation strategy based on targeted domestication of LAB and pit mud should be devised to facilitate the efficient conversion of lactic acid into caproic acid while preventing the negative effects of excessive accumulation of lactic acid. In this way, the traditional experience-based brewing process is upgraded into a data-driven precision fermentation system, promoting the continuous development of the strong-flavor Baijiu industry towards higher quality, standardization, and intelligence. Furthermore, adaptive evolution or synthetic biology approaches should be used to enhance the beneficial functions of LAB (e.g., aroma production and bacteriocin production), increase their probiotic potential, and promote their cross-disciplinary and cross-industry applications.

## Figures and Tables

**Table 1:** Major LAB in Daqu, Fermented Grains, and Pit Mud of Strong-flavor Baijiu and Their Functions.

Source	Types of LAB	Main Functions	References
Fermented Grains	*Limosilactobacillus panis*, *Limosilactobacillus fermentum*, *Acetilactobacillus acetotolerans*, *Lv. brevis*, *Companilactobacillus crustorum*, *Lt. curvatus*, *Lp. pentosus*, *Lc. garvieae*, *Lc. citreum*, *We. cibaria*, *We. confusa*, *L. acidipiscis*, *L. paracollinoides*, *Lc. lactis* et al.	Acid production drives the synthesis of flavor precursors. Through key metabolic pathways, it generates lactic acid, propionic acid, butyric acid, and other organic acids, thereby directly influencing flavor composition.	[22,24]
Pit Mud	*Ac. acetotolerans*, *Lp. pentosus*, *Ls. zeae*, *Ls. paracasei*, *Pc. acidilactici*, *B. coagulans*, *E. thailandicus*, *E. italicus*, *E. pseudoavium*, *E. casseliflavus*, *E. durans*, *L*. *camelliae*, *Ls. casei*, *Lentilactobacillus laojiaonis*, *Lc. lactis*, *Lc. garvieae*, *Lc*. *mesenteroides*, *We. paramesenteroides* et al.	The key to aroma development lies in maintaining a balanced microflora. The lactic acid produced by LAB metabolism is esterified to form ethyl lactate. Additionally, by maintaining a highly acidic environment, LAB inhibit the growth of unwanted bacteria and stabilize the microbial community.	[22,25,26,27,28,29]
Daqu	*Lc. mesenteroides*, *We. hellenica*, *Lv. brevis*, *Co. crustorum*, *Lt. curvatus*, *Lp. pentosus*, *Lc. citreum*, *We. cibaria*, *We. confusa*, *B*. *licheniformis*, *E. faecium*, *E. gallinarum*, *Lp. plantarum*, *Lb. dextrinicus*, *Pc. pentosaceus*, *Lc. lactis* et al.	Substrate conversion and flavor regulation involve the breakdown of starch and proteins and the synthesis of amino acids. These processes thereby influence the formation of higher alcohols and nitrogenous flavor compounds.	[22,28,30]

**Table 2:** Dominant LAB taxa, relative abundance, and fermentation stages in strong-flavor Baijiu brewing ecosystems.

Niche	Fermentation Stage	Dominant LAB	Relative Abundance	Testing Method	References
Fermented Grains	Early stage (0–7 days)	*Lactobacillus*, *Weissella*, *Pediococcus*	10–30%	High-throughput sequencing	[22]
	Mid-term (7–25 days)	*Lactobacillus*	50–75%	High-throughput sequencing	[22,74]
	Mid to late stage (25–60 days)	*Lactobacillus*	78–96.42%	High-throughput sequencing	[22,74]
Pit Mud	Different cellar ages	*Ls. paracasei*, *Ls. zeae*, *Pc. acidilactici*	2.43–16.19%	Culturing separately and sequencing	[25,26,28]
Daqu	Storage period	*Lc. lactis*, *Pc. pentosaceus*, *Weissella*	30–60%	Metagenome	[30,72]

## Data Availability

No new data were created or analyzed in this study. Data sharing is not applicable to this article.
